# A Multi-Point Surveillance for Antimicrobial Resistance Profiles among Clinical Isolates of Gram-Negative Bacteria Recovered from Major Ha’il Hospitals, Saudi Arabia

**DOI:** 10.3390/microorganisms9102024

**Published:** 2021-09-24

**Authors:** Kamaleldin B. Said, Ahmed Alsolami, Amany M. Khalifa, Nuha A. Khalil, Soha Moursi, Abuzar Osman, Dakheel Fahad, Ehab Rakha, Musleh Rashidi, Safia Moussa, Abdelhafiz I. Bashir, Fayez Alfouzan, Sahar Hammam, Taha E. Taha, Awdah Al-hazimi, Ahmed Al Jadani, On behalf of the Ha’il COM Research Unit Group

**Affiliations:** 1 Department of Pathology, College of Medicine, University of Ha’il, Ha’il 55476, Saudi Arabia; a.Khalifa@uoh.edu.sa (A.M.K.); n.khalil@uoh.edu.sa (N.A.K.); Sohasma2011@gmail.com (S.M.); 2 Genomics, Bioinformatics and Systems Biology, Carleton University, 1125 Colonel-By Drive, Ottawa, ON K1S 5B6, Canada; 3 ASC Molecular Bacteriology, McGill University, 21111 Lakeshore Rd, Montreal, QC H9X 3L9, Canada; 4 Department of Internal Medicine, College of Medicine, University of Ha’il, Ha’il 55476, Saudi Arabia; a.alsolami@uoh.edu.sa (A.A.); a.aljadani@uoh.edu.sa (A.A.J.); 5 Department of Pharmacology, College of Medicine, University of Ha’il, Ha’il 55476, Saudi Arabia; a.osman@uoh.edu.sa; 6 Departments of Microbiology, Education, Research and Training, King Khalid Hospital, Ha’il 55476, Saudi Arabia; daldakheel@moh.gov.sa (D.F.); ehabrakha@yahoo.com (E.R.); 7 Clinical Pathology Department, Faculty of Medicine, Mansoura University, Mansoura 35516, Egypt; 8 Ministry of Health, Hail Region, Ha’il 55476, Saudi Arabia; Mreshidi@moh.gov.sa; 9 Department of Microbiology, King Salman Specialist Hospital, Ha’il 55476, Saudi Arabia; safiamoussa89@yahoo.com (S.M.); Has-lab-kssh@moh.gov.sa (F.A.); 10 Department of Physiology, College of Medicine, University of Hail, Ha’il 55476, Saudi Arabia; ah.bashir@uoh.edu.sa (A.I.B.); dr.awdah@uoh.edu.sa (A.A.-h.); 11 Department of Microbiology, Maternity and Children Hospital, Ha’il 55476, Saudi Arabia; Sahar.hammam@gmail.com; 12 Department of Epidemiology, John Hopkins Bloomberg School of Public Health, Baltimore, MD 21205, USA; ttaha1@jhu.edu

**Keywords:** antimicrobial-resistance surveillance, nosocomial-resistance, Gram-negative bacterial resistances, multifactorial nosocomial resistances

## Abstract

The devastating nosocomial resistance is an on-going global concern. Surveillance of resistance is crucial for efficient patient care. This study was aimed to conduct a surveillance in four major Ha’il Hospitals from September to December 2020. Using a multipoint program, records of 621 non-duplicate Gram-negative cultures were tested across 21 drugs belonging to different categories. Major species were *Klebsiella pneumoniae* (*n* = 187, 30%), *E. coli* (*n* = 151, 24.5%), *Pseudomonas aeruginosa*, (*n* = 84, 13.6%), *Acinetobacter baumannii* (*n* = 82, 13.3%), and *Proteus mirabilis* (*n* = 46, 7%). Based on recent resistance classifications, *A. baumanni*, *P. aeruginosa*, and enteric bacteria were defined as pan-resistant, extremely resistant, and multi-drug resistant, respectively. *A. baumannii* (35%) and *K. pneumoniae* (23%) dominated among coinfections in SARS-CoV2 patients. The “other Gram-negative bacteria” (*n* = 77, 12.5%) from diverse sources showed unique species-specific resistance patterns, while sharing a common Gram-negative resistance profile. Among these, *Providencia stuartii* was reported for the first time in Ha’il. In addition, specimen source, age, and gender differences played significant roles in susceptibility. Overall infection rates were 30% in ICU, 17.5% in medical wards, and 13.5% in COVID-19 zones, mostly in male (59%) senior (54%) patients. In ICU, infections were caused by *P. mirabilis* (52%), *A. baumannii* (49%), *P. aeruginosa* (41%)*, K. pneumoniae* (24%)*,* and *E.* *coli* (21%), and most of the respiratory infections were caused by carbapenem-resistant *A. baumannii* and *K. pneumoniae* and UTI by *K. pneumoniae* and *E.* *coli.* While impressive IC, hospital performances, and alternative treatment options still exist, the spread of resistant Gram-negative bacteria is concerning especially in geriatric patients. The high selective SARS-CoV2 coinfection by *A. baumannii* and *K. pneumoniae*, unlike the low global rates, warrants further vertical studies. Attributes of resistances are multifactorial in Saudi Arabia because of its global partnership as the largest economic and pilgrimage hub with close social and cultural ties in the region, especially during conflicts and political unrests. However, introduction of advanced inter-laboratory networks for genome-based surveillances is expected to reduce nosocomial resistances.

## 1. Introduction

The emergence and spread of antimicrobial resistance are one of the major global issues currently threatening the health and wealth of nations and a major cause of morbidity, mortality, and healthcare costs for decades [1,2,3]. Because of this, the World Health Organization (WHO) has declared the antimicrobial resistance as a public health threat and urged countries to develop surveillance programs [4]. For instance, according to the US Center for Disease Control and Prevention, nearly 1.7 million hospitalized patients acquire healthcare-associated infections (HAIs) annually, and more than 98,000 patients (i.e., 1 in 17) die due to these [5]. Similarly, the European Center for Disease Prevention and Control published the results of two point-prevalence surveys of HAIs and antimicrobial use in hospitals and long-term facilities. With 8.9 million infections, 33,000 deaths, and EUR 1 billion in annual healthcare cost, they called for action to address the problem [6,7,8]. In the Middle East, updated reports on surveillance database of HAIs and resistances are limited or outdated; however, countries like Saudi Arabia are leading in advanced healthcare systems.

Several initiatives have been developed worldwide to combat HAI and drug resistances. Hays et al. (2019) [9], on behalf of the European Joint Programming Initiative on Antimicrobial Resistance Transnational Working Group ‘Antimicrobial Resistance—Rapid Diagnostic Tests’ (JPIAMR AMR–RDT), proposed a point-of-care testing (PoCT) initiative for rapid infectious disease and antimicrobial resistance PoCT in the fight against increasing antimicrobial resistance. Furthermore, updated databases of surveillance in Europe have been developed and made available at regional and international settings to ease communication between scientists [10,11]. More advanced interventions have integrated molecular tools along with surveillance. Evidence from three large observational trials using a prediction model applied to predict cost and medical outcomes for thousands of sepsis patients in seven hospitals have indicated that rapid PCR identification of microorganisms has the potential to become a cost-effective component for managing infections [12]. Thus, the magnitude of the Gram-negative resistance problem as well as the profiles of the endemic bacterial strains and their antibiogram are unknown in the region due to the lack of frequent surveillance systems.

With advances in healthcare systems, significant gaps are created in understanding the epidemiology and pathogenicity of the new agents. A comprehensive study has identified some of these gaps and proposed a road map for research [13]. One of the major two issues identified was the current variation in surveillance definitions of hospital-associated infections. The Centers for Disease Control and Prevention’s (CDC) National Healthcare Safety Network (NHSN) recently revised a number of surveillance definitions to address many of these limitations. This included definitions of infections occurring in long-term care facilities as described in the document CDC/National Healthcare Safety Network Surveillance Definitions for Specific Types of Infections (available at https://www.cdc.gov/nhsn/pdfs/pscmanual/17pscnosinfdef_current.pdf, accessed 20 February 2021 [14]). The second pressing issue identified was the lack of understanding of pathogenesis and epidemiology of these infections, especially those of emerging multidrug-resistant (MDR) organisms.

Infectious agents and their susceptibility patterns have been found to vary from one regional setting to another; a fact which highlights the importance of having local surveillance data for planning and implementing infection prevention and control measures [15,16,17]. High-income countries, including the United States, have managed to decrease the burden of HAIs by 30% through effective implementation of surveillance systems [18]. Similarly, in Europe, updated databases of surveillance have been developed and made widely available at regional and international settings to ease communication between scientists. In the Middle East, there is a need for active surveillance programs and updated online data management systems and communication platforms. However, in Saudi Arabia, many recommendations are being made to combat new and emerging infectious diseases. Baraka et al. (2019) [19] reported on the need for training and educating health care workers about AMR in the Eastern Saudi Arabia. In the light of the annual Hajj vaccine requirements and possible new challenges, it has been imperatively recommended to continue surveillance of antimicrobial resistance [20]. Furthermore, a pilot multicenter study in intensive care units in five cities from the Kingdom of Saudi Arabia implementing the International Nosocomial Infection Control Consortium’s (INICC) Multidimensional Approach (IMA) has shown a significant reduction in the rates of Central Line-associated Bloodstream Infections (CLABSI) in the ICUs [21]. In another report, tuberculosis rates varied significantly from one region to another, as shown by the results from the first representative national anti-TB drug resistance survey in Saudi Arabia. This study suggested that the proportion of MDR TB was relatively low in the country, despite a higher primary drug resistance [22]. In addition, a study analyzing 3404 samples by culture-based and molecular techniques found only 2% prevalence of suspected tuberculosis in Ha’il province while Riyadh and Dammam had the highest prevalence, 22% and 21%, respectively, at the national level in Saudi Arabia [23].

Multi-point surveillances of nosocomial infections, dominant types, and resistance profiles in Ha’il healthcare facilities have not been well documented or are outdated if any. The incidence rates in 2331 wound infections and the most effective antibiotics used were studied in three different departments in a major hospital in the Ha’il province [24]. The antibiogram patterns of *Escherichia coli, Pseudomonas aeruginosa*, and *Klebsiella* indicated occurrence of significant changes in over two decades time. The aforementioned multicenter study reported in [21] examined a single-point survey focusing on the impact of International Nosocomial Infection Control Consortium’s (INICC) multidimensional approach on rates of central line-associated bloodstream infections and pneumonia in intensive care units in Saudi Arabia. A multi-university theoretical study in Saudi Arabia described nursing students’ perception of the infection prevention climate of their training and provided unique perspective on factors that affect the infection prevention climate [25].

It has been well established that extended-spectrum cephalosporin- and carbapenem-resistant Gram-negative bacilli are widely disseminated in humans, animals, and the environment worldwide. Recent studies suggested steady increases in emerging resistant enteric bacterial species in healthcare settings. For these reasons, many global recommendations were made that emphasized the need for research activity to understand the epidemiology and pathogenicity of the newly emerging strains [26]. Carbapenemase production in carbapenem-resistant Gram-negative bacteria isolated from hospitalized patients in Saudi Arabia was reported [27]. The results showed dominance of OXA-48, NDM-1, and VIM-4 enzymes and the first report of OXA-72 and NDM-1 in *A. baumannii* in the country. Although some vertical studies on genetic profiling have been reported, local studies on the incidence and prevalence rates of these infections are needed in the region. Zoonotic and reverse zoonotic transmissions have been increasingly reported. In the Middle East, the current elevation in population dynamics is a significant contributing factor in the spread of resistances in the region. In hospitals, carbapenemases producers appear to be dominant, while extended-spectrum beta-lactamases (ESBL) and colistin resistance are becoming serious problems in animals due to the continuous use of colistin in veterinary medicine [28]. Genetic profiling of carbapenem-resistant *K.* *pneumoniae* isolates from a tertiary care hospital in Saudi Arabia suggested clonal diversity within the dominant outbreak strain carrying polyclonal OXA-48 gene carbapenemase [29,30]. Thus, emerging infections, particularly those caused by zoonotic and reverse zoonotic strains with increased virulence in communities, are significant risks to public health.

## 2. Materials and Methods

### 2.1. Ha’il City and All Its Socio-Economic Strata

Ha’il province has a total population of about one million. It is in the north-central Saudi Arabia, bordering five provinces, namely, Madinah, Tabouk, Northern Border, Riyadh and Qassim. The capital of Ha’il is the city of Ha’il, which lies in the Waadi Ha’il surrounding the Shammar mountain ranges. Ha’il’s magnificent prehistoric rock carving and archaeological excavations are major attraction sites for tourists. The region has four major hospitals: King Salman Specialist Hospital and King Khalid Hospital, which are tertiary care centers; Ha’il General Hospital (245 beds); and Maternity and Children Hospital (300 beds). The four hospitals primarily serve Ha’il City and all socioeconomic strata of the province. In addition, there are many private hospitals and clinics in the city; however, most of the specimens are collected and processed at King Khalid Hospital for non-specialized procedures.

### 2.2. Study Designs, Data Sources, and Statistical Analysis (From September to December 2020)

The goal of this comprehensive work was to survey antimicrobial resistances and all factors involved which would help in establishing and/or strengthening a platform for efficient stewardship program in all participating hospitals and clinics. We investigated a retrospective cross-sectional study of positive culture reports of all Gram-negative non-duplicate isolates collected from four major cohort Ha’il hospitals between September and December 2020. Data were obtained from microbiology laboratory records, hospital medical records, COVID-19 isolation zones, different hospital wards, as well as from various sources within hospitals. The data from the four participating hospitals included but were not limited to resistance profiles, SARS-CoV2 bacterial coinfections, specimen types and collection sites, admitting wards, and age and gender differences. Collected data were analyzed using the Statistical Package for Social Sciences (SPSS; Version 23 SPSS version 23.0 for Windows (SPSS, Inc., Chicago, IL, USA)) software. The microbiology laboratories in the hospitals are equipped with advanced systems and highly skilled laboratory technicians working under close supervision of specialized MD microbiologists. Pathogens were identified using routine standard bacteriological methods and ID and susceptibility testing using automated systems. This included primarily GeneXpert systems for molecular diagnostics and strain and species confirmations. In addition, the Vitek 2 system (bioMérieux, Marcy-l’Étoile, France), BD Phoenix system (BD Biosciences, Franklin Lakes, NJ, USA), MicroScan plus (Beckman Coulter, Brea, CA, USA), and BD BACTEC system (BD Biosciences) were used for the identification and antimicrobial sensitivity analysis of microorganisms. These systems are used in different hospitals; however, all isolates were screened by the BD Phoenix combined identification, antimicrobial testing, and fluorescence control at King Khalid Hospital. Routine bacteriology work was applied for confirmation and vertical analysis. Susceptibility was confirmed by culture and agar diffusion experiments. The susceptibility testing and breakpoint interpretive standards were carried out in accordance to the recommendations of the Clinical and Laboratory Standard Institute (CLSI document M100S-26) [19].

### 2.3. Classification as Multi-Resistant, Extremely-Resistant, and Pan-Resistant Types (MDR, XDR, PDR)

This study included data from hospitalized patients at different points of care. Microorganisms were classified as MDR, XDR, and PDR as defined in the guidelines of the European Centre for Disease Control [20]. The MDR type was defined as having acquired non-susceptibility to at least one agent in three or more antimicrobial categories, XDR was defined as non-susceptible to at least one agent in all but two or fewer antimicrobial categories (i.e., bacterial isolates remain susceptible to only one or two categories) and PDR was defined as non-susceptibile to all agents in all antimicrobial categories [31].

## 3. Results

The total number of records screened at each hospital point was over 3000 positive culture isolates in four months; however, all duplicate isolates, especially in extended stay units, and tests that did not cover all drug categories, were removed. This decreased the precise number of isolates included in the study to 621.

The total number of records screened at each hospital point was over 3000 positive cultures isolates in four months; however, all duplicate isolates, especially in extended stay units, and those that did not cover all drug categories, were removed. This made the precise number of isolates to 621 that were included in the study.

### 3.1. Kleb. pneumoniae (MDR)

As shown in Table 1, a total of 178 clinical specimens from different wards and departments in participating hospitals were positive for *Kleb. pneumoniae* isolates. Of these, 42 (24%) were from ICU specimens alone, which were mostly urine, followed by sputum, blood, and wound. An almost equal rate of isolation was observed in the COVID-19 ward specimens (23%), where specimen types were the same, namely, blood, sputum, urine and wound. The specimens were also the same in surgical and medical wards, but with slightly lower rates of isolations (14% and 12%, respectively). However, an isolation frequency of 24% was reported in specimens from all other sources. Frequencies of isolation increased with age; infection rates in youths, adults, and seniors were 11%, 33%, and 57%, respectively. Infection rates in males (53%) were higher than those reported in females (43%).

**Figure 1:**
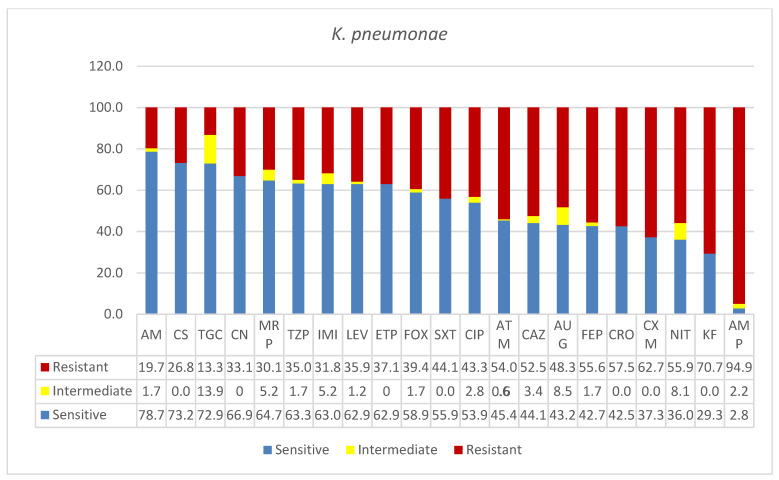
Antimicrobial sensitivity patterns of *K. pneumoniae* isolates to 21 antibiotics. Abbreviations (in the order in which they appear in the figure): AK, amikacin; CS, colistin; TGC, tigecycline; CN, gentamicin; MRP, meropenem; TZP, tazobactam; IMI, imipenem; LEV, levofloxacin; ETP, ertapenem; FOX, cefoxitin; SXT, trimethoprim*/sulfamethoxazole; CIP, ciprofloxacin; ATM, aztreonam; CAZ, ceftazidime; AUG, amoxicillin*/clavulanic acid (2/1); FEP, cefepime; CRO, ceftriaxone; CXM, cefuroxime; NIT, nitrofurantoin; KF, cephalothin; AMP, ampicillin.

As shown in Figure 1, *K. pneumoniae* was classified as MDR in this study. Over 50% resistance was seen among the isolates, and the percentages were higher for cephalothin (KF, 70.7%) and ampicillin (AMP, 94.9%). The carbapenems showed similar resistances: ertapenem (ETP, 37%), imipenem (IMI, 31.8%), and meropenem (MRP, 30%). Similarly, the following antibiotics showed similar resistances in the narrow range between 30 and 40%: amoxicillin*/clavulanic (AUG, 48.3%), ciprofloxacin (CIP, 43.3%), trimethoprim*/sulfamethoxazole (SXT, 44%), cefoxitin (FOX, 39.4%), levofloxacin (LEV, 39.5 %), piperacillin/tazobactam (TZP, 35%), gentamicin (CN, 33%), tigecycline (TGC, 13%), colistin (CS, 26.8%), and amikacin (AK, 19.7). *K. pneumoniae* showed many intermediate resistances to a wide range of antibiotics tested in this study.

### 3.2. E. coli (MDR)

**Figure 2:**
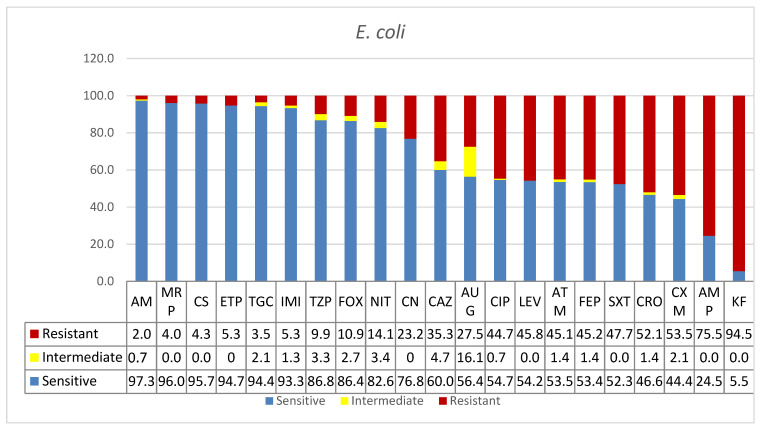
Antimicrobial sensitivity patterns of *E. coli* isolates to 21 antibiotics. Abbreviations (in the order in which they appear in the figure): AK, amikacin; MRP, meropenem; CS, colistin; ETP, ertapenem; TGC, tigecycline; IMI, imipenem; TZP, piperacillin/tazobactam; FOX, cefoxitin; NIT, nitrofurantoin; CN, gentamicin; CAZ, ceftazidime; AUG amoxicillin*/clavulanic acid (2/1); CIP, ciprofloxacin; LEV, levofloxacin; ATM, aztreonam; FEP, cefepime; SXT, trimethoprim*/sulfamethoxazole; CRO, ceftriaxone; CXM, cefuroxime; AMP, ampicillin; KF, cephalothin.

One hundred and fifty-one clinical isolates of *E. coli* were included in this study. As shown in Table 1, the majority of the isolates were from the ICU (*n* = 31, 21%), where urine was the most frequently positive specimen for *E. coli* (*n* = 16). This was followed by equal numbers of isolations from sputum and blood (*n* = 6 each) and 3 isolates from wound infections. Only 5% (*n* = 7) of *E. coli* isolates were from COVID-19 patients. Nineteen isolates (13%) were reported from surgical ward specimens of mostly urine and wound infections. However, the highest number of isolates came from medical wards (*n* = 44; 29%), over half of these being found in urine specimens (*n* = 26). The infection rates amongst young, adult, and senior patients were 26%, 32%, and 42%, respectively, in contrast to the overall bacterial infection rates reported in this study (16%, 30%, and 54%). As shown in Table 1, infection rate in male patients was 43% compared with 57% in females. However, the overall rate of bacterial infection in males was 59% and 41% in females. As shown in Figure 2, most of the antibiotics tested were effective against *E. coli* except for a few; namely, cephalothin (KF) and ampicillin (AMP), which were 94.5% and 75.5% resistant, respectively. Wide ranges of antibiotics were effective in the treatment of *E. coli.* These included amikacin (AK, 97.3%), meropenem (MRP, 96%), colistin (CS, 95.7%), ertapenem (ETP, 94.7%), tigecycline (TGC, 94.4%), imipenem (IMI, 93.3%), piperacillin/tazobactam (TZP, 86.8 %), cefoxitin (FOX, 86.4%), nitrofurantoin (NIT, 82.6%), and gentamicin (CN 76.8%), in decreasing order of effectiveness.

### 3.3. Pseudomonas aeruginosa (XDR)

**Figure 3:**
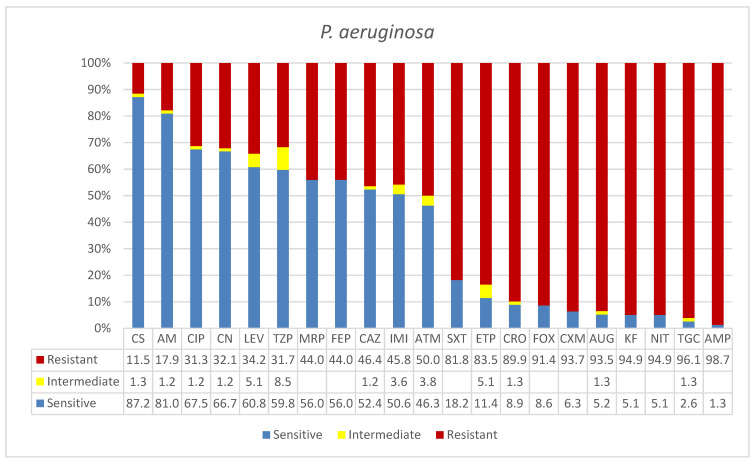
Antimicrobial sensitivity patterns of *P. aeruginosa* isolates to 21 antibiotics. Abbreviations (in the order in which they appear in the figure): CS, colistin; AK, amikacin; CIP, ciprofloxacin; CN, gentamicin; LEV, levofloxacin; TZP, piperacillin/tazobactam; MRP, meropenem; FEP, cefepime; CAZ, ceftazidime; IMI, imipenem; ATM, aztreonam; SXT, trimethoprim*/sulfamethoxazole; ETP, ertapenem; CRO, ceftriaxone; FOX, cefoxitin; CXM, cefuroxime; AUG, amoxicillin*/clavulanic acid (2/1); KF, cephalothin; NIT, nitrofuran; TGC, nitrofurantoin tigecycline; AMP, ampicillin.

Eighty-four clinical isolates of *P. aeruginosa* were reported from different wards and units (Table 1). The majority of the positive specimens were reported at ICU (41%) where most isolation were made from sputum (*n* = 15) followed by urine and blood with equal isolates from each (*n* = 5). The surgical, medical, and AKU—AMR wards units reported 11% positive specimens from the former ward and 7% each for the latter two. The age-specific infection rates were 23% in young patients, whereas the rates were similar in adult and senior age groups (39% and 38%, respectively). The overall rates of Gram-negative bacteria for these three groups were 16%, 30%, and 54%, respectively, which were different from the overall bacteria rates. Gender-specific infection survey showed 75% of men acquired *P. aeruginosa* compared with 25% of women (Table 1), in contrast to 59% and 41% for these two groups, respectively. As shown in Figure 3, *P. aeruginosa* was one of the organisms that showed high levels of resistance to routine drugs used for treatment, including carbapenems. This included 10 drugs with over 80% resistances, namely: ampicillin (AMP, 98.7%), tigecycline (TGC, 96%), nitrofuran (NIT, 94.9%), cephalothin (KF, 94.4%), amoxicillin*/clavulanic acid (2/1) (AUG, 93.5%), cefuroxime (CXM, 93.7%), cefoxitin (FOX, 91.4), ceftriaxone (CRO, 89.9%), ertapenem (ETP, 83.5), and trimethoprim*/sulfamethoxazole (SXT, 81.8%). Antibiotics with higher sensitivities were: colistin (CS, 87%), amikacin (AK, 81%), ciprofloxacin (CIP, 67.5%), gentamicin (CN, 66.7%), levofloxacin (LEV, 60.8%), and piperacillin/tazobactam (TZP, 59.8%). *P. aeruginosa* showed resistances over 50% to carbapenems; it was 83.5% in the case of ertapenem (ETP).

### 3.4. Acinetobacter baumannii (PDR)

**Figure 4:**
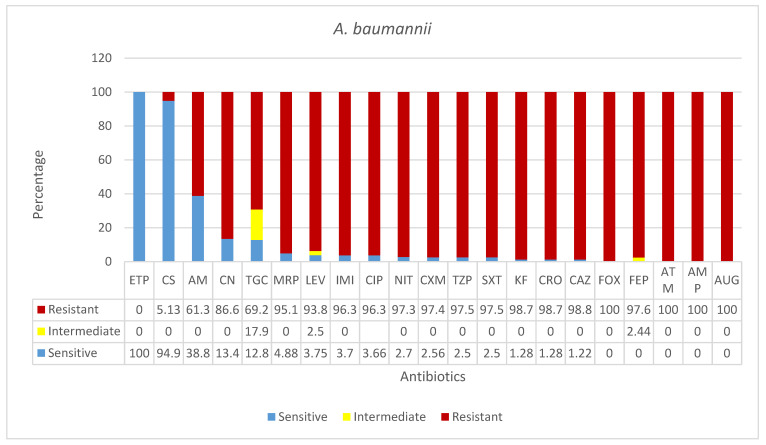
Antimicrobial sensitivity patterns of *A. baumannii* isolates to 21 antibiotics. Abbreviations (in the order in which they appear in the figure): ETP, ertapenem; CS, colistin; AK, amikacin; CN, gentamicin; TGC, tigecycline; MRP, meropenem; LEV, levofloxacin; IMI, imipenem; CIP, ciprofloxacin; NIT, nitrofurantoin; CXM, cefuroxime; TZP, piperacillin/tazobactam; SXT, trimethoprim*/sulfamethoxazole; KF, cephalothin; CRO, ceftriaxone; CAZ, ceftazidime; FOX, cefoxitin; FEP, cefepime; ATM, aztreonam; AMP, ampicillin; AUG amoxicillin*/clavulanic acid (2/1).

As shown in Figure 4 and Table 1, in this study, *A.* *baumannii* was isolated from 82 clinical specimens collected in all participating hospitals. An overwhelming majority of isolates were found in respiratory samples in ICU and in blood from COVID-19 patients (49% and 35%, respectively; Table 1). The *A.* *baumannii* infection rates in young (9%) and adult (27%) categories of patients were similar to the overall bacterial infection rates in this study; however, higher percentage than the overall rate was reported in the case of seniors (64%). Infection rate in men (59%) was higher than the one in women (41%). In this study, *A.* *baumannii* was found to be the most resistant pathogen isolated from clinical specimens in all four hospitals. Isolates of this species were fully resistant to almost all antibiotics tested, except for amikacin (AK, 61.25%), colistin (CS, 5%), and ertapenem (ETP, 0%). The latter antibiotic was 100% effective in the treatment of this infection, while 69.23% of *A.* *baumannii* isolates were resistant to tigecycline (TGC), only 12.82% were sensitive to it, and 17.95% had a relatively higher intermediate response.

### 3.5. Proteus mirabilis (MDR)

**Figure 5:**
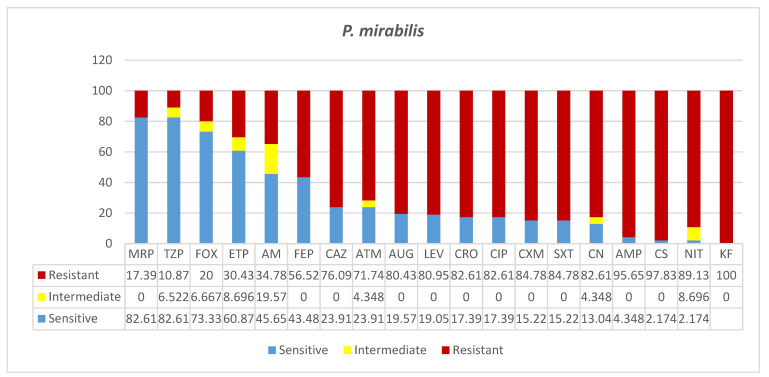
Antimicrobial sensitivity patterns of *P. mirabilis* isolates to common antibiotics Abbreviations (in the order in which they appear in the figure): MRP, meropenem; TZP, piperacillin/tazobactam; FOX, cefoxitin; ETP, ertapenem; AK, amikacin; FEP, cefepime; CAZ, ceftazidime; ATM, aztreonam; AUG, amoxicillin*/clavulanic acid (2/1); LEV, levofloxacin; CRO, ceftriaxone; CIP, ciprofloxacin; CXM, cefuroxime; SXT, trimethoprim*/sulfamethoxazole (1/19); CN. gentamicin; AMP, ampicillin; CS, colistin; NIT, nitrofurantoin; KF, cephalothin.

Forty-six clinical isolates of *P. mirabilis* were reported in this study. Of these, 52% were found in ICU specimens, almost equally frequently isolated from blood, sputum, urine, and wound specimens (Table 1). While few isolations occurred in surgical and COVID-19 wards, 28% of them were found in the medical ward specimens, mostly urine samples. In the case of *P. mirabilis* infection, the majority of patients (83%) were seniors, and only 17% were adults, in contrast to the overall infection rates of 16%, 30%, and 54% in youths, adults, and seniors, respectively. *P. mirabilis* infection rates in men (66%) were slightly over two-fold higher than that in females (34%). To all the antibiotics tested, a certain degree of resistance, ranging from 82% to 100% was reported (Figure 5). To four antibiotics, almost 100% of *P. mirabilis* isolated were resistant, namely, to cephalothin (KF, 100%), nitrofuran (NIT, 89%), colistin (CS, 97.8%), and ampicillin (AMP, 95.7%). The bacteria showed similar rates of resistance to nine antibiotics: aztreonam (ATM, 72%), ceftazidime (CAZ, 76%), amoxicillin*/clavulanic acid (2/1) (AUG, 80.4%), levofloxacin (LEV, 80.95%), ceftriaxone (CRO, 82.6%), ciprofloxacin (CIP, 82.6%), gentamicin (CN, 82.6%), cefuroxime (CXM, 84.8%), and trimethoprim*/sulfamethoxazole (1/19) (SXT, 84.8%). Significant resistances and increasing intermediate resistances were seen in the case of the antibiotics of choice, including carbapenems: meropenem (MRP, 17.4%), piperacillin/ tazobactam (TZP, 10.9%), cefoxitin (FOX, 20%), ertapenem (ETP, 30.4%), amikacin (AK, 34.8%), and cefepime (FEP, 56.5%).

### 3.6. Other Gram-Negative Bacteria (MDR)

All the rest of the Gram-negative bacterial isolates (71 isolates) was included in this category due to low, non-significant rates of reporting. The details of age, gender, specimen, and antibiograms are shown in Table 2. These species were isolated at low rates from various types of specimens. As shown in Table 1, only three COVID-19 coinfections were reported in this group. The ICU, surgical, and medical wards reported 21%, 18%, and 23% of other Gram-negative bacteria isolation rates, respectively. In addition, low rates of isolations were reported from diverse sources, altogether representing 34% of the Gram-negative isolates. For the young and adult groups, similar rates were reported (20% and 21%, respectively), while three times higher frequency of isolation was found in seniors (61%). The overall isolation rates from youths, adults, and seniors were 16%, 30%, and 54%, respectively. Infection rates were higher in males (63%) than females (37%) (Table 1 and Table 2).

**Figure 6:**
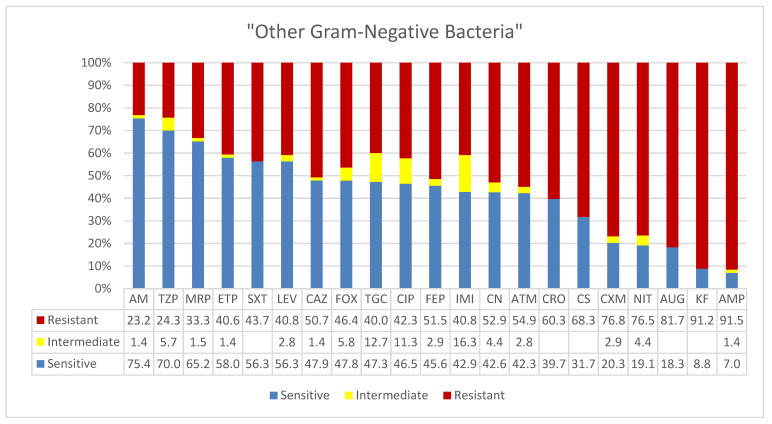
Antimicrobial sensitivity patterns of isolates of “other Gram-negative bacteria” to 21 antibiotics. Abbreviations (in the order in which they appear in the figure): AK, amikacin; TZP, piperacillin/tazobactam; MRP, meropenem; ETP, ertapenem; SXT, trimethoprim*/sulfamethoxazole; LEV, levofloxacin; CAZ, ceftazidime; FOX, cefoxitin; TGC, tigecycline; CIP, ciprofloxacin; FEP, cefepime; IMI, imipenem; CN, gentamicin; ATM, aztreonam; CRO, ceftriaxone; CS, colistin; CXM, cefuroxime; NIT, nitrofurantoin; AUG, amoxicillin*/clavulanic acid (2/1); KF, cephalothin; AMP, ampicillin.

High levels of intermediate resistance were reported for these sporadic isolates. As shown in Figure 6, these isolates showed almost identical patterns of susceptibilities, resistances, and intermediate resistances across the antibiotics tested. Six antibiotics showed effectiveness in over 50% of isolates, namely, amikacin (AM, 75.4%), piperacillin/tazobactam (TZP, 70%), meropenem (MRP, 65%), ertapenem (ETP, 58%), trimethoprim*/sulfamethoxazole (SXT, 56.3%), and levofloxacin (LEV, 56.3%). Similarly, to six other antibiotics the isolates were intermediately resistant: cefoxitin FOX, tigecycline (TGC), ciprofloxacin (CIP), cefepime (FEP), imipenem (IMI), and gentamicin (CN). However, to nine antibiotics, they showed resistance rates ranging from 50 to over 90%: ampicillin (AMP, 91.5%), cephalothin (KF, 91.2%), amoxicillin*/clavulanic acid (2/1) (AUG, 81.7%), nitrofuran (NIT, 76.5%), cefuroxime (CXM, 76.8%), colistin (CS, 68%), ceftriaxone (CRO, 60.3%), aztreonam (ATM, 54.9%), gentamicin (CN, 52.9%). Isolates of a single species showed similar patterns of intra-species antimicrobial resistances in addition to cross-species pan-resistances. For instance, *P. stuartii* isolates showed patterns of high imipenem, cephalosporin, and colistin resistance. These isolates were also resistant to cephalothin, gentamicin, colistin, nitrofuran, ampicillin, and potentiated ampicillin, which was common to all species reported in this category. Similarly, *Sten. maltophilia* showed a distinct pattern of resistance to imipenem and cephalosporins in addition to the aforementioned common resistances.

## 4. Discussion

In this study, *Klebsiella pneumoniae* showed the highest frequency of isolation (*n* = 178) from diverse clinical specimens. These were comprised of ICU specimens primarily from urinary tract infections and COVID-19 wards (24% and 23%, respectively). Infection rates were proportional to the increase in age in a fixed pattern of increasing increment. In young patients, the rate was 11%, while in adults and seniors it was 33% and 57%, respectively. This is similar to a retrospective cross-sectional study on the most common UTI-causative organisms associated with the emergence of antimicrobial resistance in Riyadh, Saudi Arabia, where *K. pneumoniae* (15%) ranked second to *E. coli* (52%) as the most common uropathogen. Despite the differences in study designs and the socio-economic strata of Riyadh and Ha’il cities, the aforementioned and the current study both agreed on the devastatingly high rates of resistances among clinical isolates of the two species [32]. Depending on the local hospital and sample size, the rates of isolations of *K. pneumoniae* as a uropathogen have slightly varied across many countries and continents. In China, a recent study showed dominance of *E. coli* in urine (35.27%) while *P. aeruginosa* (20.67%) and *K. pneumoniae* (13.99%) dominated in sputum samples (Yang and Ji 2020). Similar patterns have been reported in pediatric (<15 years) bacterial infection cases in Africa, where *E. coli* (49.5%) and *Klebsiella* (27.9%) were dominant [33]. Concerns over a wide spread of resistant Gram-negative bacteria in the Middle Eastern countries and their potential origins have been reported [28]. Finally, the low resistance to colistin, which commonly indicates transfer from livestock animals, accompanied by a high resistance to ampicillins in *K. pneumonia*, similar to that of *E. coli*, implied nosocomial resistance. Antibiotic misuse in the community, agricultural and animal productions may also have played a role. Similar to our findings, a recent surveillance of bloodstream infections has also identified the same types of species with similar antibiogram patterns in the Aljouf region of Saudi Arabia [34]. However, the resistances reported in a four-year surveillance in Madina, Saudi Arabia, [35] were slightly higher than those in the present study, reflecting the study duration and sample and city sizes.

*Klebsiella pneumonia* isolates were completely resistant to almost all antibiotics tested, except for TGC, AK, CS, and ETP, to which 69.23%, 61.25%, 5%, and 0% of isolates were resistant, respectively. In addition, 17.95% of isolates had a relatively higher intermediate response to tigecycline (TGC). This antibiogram pattern is consistent with the devastating global epidemiology of multidrug-resistant *A. baumannii* that has been observed for nearly a decade [36]. However, typical of the widely known international profile of ICU infections [37], colistin and ertapenem are still the drugs of choice in the treatment of *A. baumannii* infections in our hospitals, with over 95% and 100% effectiveness, respectively. Nevertheless, the high levels of resistances to other carbapenems, imipenem (IMI, 96.3%) and meropenem (MRP, 95%), is worrisome because dominance of OXA-48, NDM-1, and VIM among Enterobacteriaceae in the region has been reported [27,28,38].

In this study, *E. coli* was the second dominant nosocomial Gram-negative species isolated from clinical specimens (*n* = 151 isolates). Most of these positive specimens were from medical wards (29%), ICU (21%), surgical wards (13%), in addition to many diverse sources (30%). Urinary tract infection was the most common in all the patients at different wards, followed by respiratory infections. The steady increase in infection rates with patient age and hospitalization—26%, 32%, and 42% in youths, adults, and seniors, respectively—is consistent with nosocomial spread among risk groups. Most of these groups were composed of females with UTI (57% compared with 43% of males). This was in sharp contrast to the overall gender-specific infection rates reported in this study (male 59%, females 41%) and to the bloodstream infection rates (male 60%, female 40%) reported in Saudi Arabia recently [34]. More important was the prevalence of *E. coli* coinfection in COVID-19 patients, which will be discussed latter, with the overall infection rates and their influence in aggravating COVID-19 and underlying diseases.

As shown in Figure 2, most of the antibiotics tested were effective against *E*. *coli*, except for cephalothin (KF) and ampicillin (AMP), to which 94.5% and 75.5% of *E*. *coli* isolates showed resistance, respectively. This pattern of resistance seems to be common globally in many regions. However, despite the potential for increasing resistances and continued warnings, many countries still administer these antibiotics, including, for instance, Turkey, Australia, and Ireland [39,40,41]. In this report, a significant number of cases were urinary tract infections in young age patients. The global prevalence of resistance to commonly prescribed antibiotics in children with urinary tract infections (UTI) caused by *E. coli* is reported to be high. This is particularly true in countries outside the Organization for Economic Co-operation and Development (OECD) [42]. Although Saudi Arabia is a member state in the OECD convention, its strategic position as an economic, social, and religion hub in the region would be one of the factors contributing to the burden of transmission of resistances. However, the high levels of colistin sensitivity and the free ranching style practiced in large animal livestock productions in Saudi Arabia would imply nosocomial resistances rather than colistin resistance transfer from food animals [26]. Furthermore, while effectiveness of carbapenems against *E. coli* UTI in Ha’il hospitals is encouraging, the appearance of intermediate resistance and the increased resistance in other enteric bacteria calls for stricter measures and evidence-based prescribing to limit transmission of resistant strains.

The hardy environmental organism *P. aeruginosa* was isolated from 84 clinical specimens. The high frequency of isolations from sputum reported at ICU (42%) in mostly overage males is consistent with the nosocomial spread in compromised patients [43]. Furthermore, there were high levels of resistances, i.e., >90% to 100%, to common hospital drugs, such as carbapenems, ampicillin, tigecycline, nitrofuran, cephalothin, cefuroxime, and potentiated amoxicillin*/clavulanic acid (2/1). This pattern, along with the effectiveness of colistin (CS, 87%), would imply a significant hospital-acquired multidrug resistance rather than a transfer from animal production practices. Even though the city of Ha’il is an environmentally friendly region, the resistance pattern showed by *P. aeruginosa* does not indicate colistin resistance transfer from the farms and environment. A recent surveillance of resistant pathogens in Egypt, Sudan, and Saudi Arabia revealed the ratios of the multidrug-resistant strains as 74.4%, 90.1%, and 97.5%, respectively. *E. coli* and *K. pneumoniae* were the most resistant to macrolides followed by penicillins and cephalosporin, while *P. aeruginosa* showed the highest resistance to penicillins followed by classes that varied among different countries [44]. Nevertheless, this potent opportunistic pathogen has a common virulence and adaptation regulatory system that operates in environmental and clinical settings. For instance, strains efflux quinolones, which are synthetic antibiotics not expected in the environment, use alkanes (oil hydrocarbons) as a carbon source, contain multidrug resistance determinants, and are capable of invading epithelial cells. All these processes are regulated by quorum-sensing and type III secretion systems [45]. The universality of these systems has been further confirmed using genomic analysis of clinical and environmental isolates that indicated a single genomic population with high phenotypic diversity [46]. Thus, it is difficult to efficiently assign genotypes to different ecosystems. It is well known that this organism has highly dynamic elements for an extended spectrum of β-lactamases resistances and overexpression of genes encoding efflux pumps. However, β-lactamase overproduction is not consistent as a mechanism involved in carbapenem resistance because the latter was effective in over 50% of isolates in this study. This warrants a vertical genetic analysis of these isolates in the future.

*Acinetobacterbaumannii* was positive in 82 clinical specimens from all participating hospitals. An overwhelming majority of these were from respiratory samples from ICU and bloody secretions from COVID-19 patients (49% and 35%, respectively) (Table 2). Surprisingly, *A.* *baumannii* coinfection rate (35%) during COVID-19 was the highest among all bacterial infections in this study. This is particularly surprising because global SARS-CoV2 coinfection rates were found to be lower compared with SARS-CoV1, MERS, and influenza outbreaks. Many coinfection studies ranked *A. baumannii* as sixth or lower in the list among other bacteria [47]. While coinfections during the COVID-19 outbreak are significantly under reported worldwide, most of the original studies (eight) were reported in China [48,49,50,51,52,53,54,55]. Only a few in the USA [56,57] one in each Singapore [58] and Italy [59]. The study population ranged from 21 to 5700 cases. While none in Singapore had a secondary infection, 50% of nonsurviviors had it in China. In addition, in Spain, a co-infection with the COVID-19 diagnosis was uncommon [60]. In the early stages in Wuhan, only 16% of hospitalized patients had secondary infections [61], and it was higher among the nonsurvivors than the survivors (50% versus 1%). The latter two groups had significant differences in white blood cell counts, absolute values of lymphocytes, platelets, albumin, total bilirubin, blood urea nitrogen, blood creatinine, myoglobin, cardiac troponin, C-reactive protein (CRP), and interleukin-6 (IL-6). Since community-acquired co-infection at COVID-19 diagnosis is new and uncommon, many decisions on combination empirical therapy were made with limited clinical experience and scientific evidence. In addition, combination therapy was largely based on previous experiences with coinfections with common respiratory pathogens, such as *Streptococcus pneumoniae* and *Staphylococcus aureus*, and viral and fungal species were not uncommon, which resulted in poor prognosis during influenza pandemics [62,63,64,65]. Similarly, in the UK, a retrospective large cohort study revealed low frequency of bacterial coinfection in early COVID-19 hospital presentation with no evidence of concomitant fungal infection [66]. Thus, the mechanisms underlying increased *A. baumannii* coinfection during SARS-CoV2 are not clear, justifying further study. *Acinetobacter baumannii* infection rates in young (9%) and adult (27%) categories were similar to the overall bacterial infection rates in this study. However, infection rates in senior patients were higher than the overall rates of bacterial infections (64%). Although old age is an established risk factor for this organism, vertical investigation of all factors involved has not been fully studied. Lethal and non-invasive pneumonia mouse model in aged mice showed higher mortality rates compared with young mice. These were accompanied by increased bacterial burdens, more severe lung injury, reduced immunity, lower efficacy of imipenem/cilastatin, and diminished vaccine efficacy [67,68]. Furthermore, gender differences in susceptibility showed that infection rates in men (59%) were higher than those in women (41%). Similarly, there is no experimental explanation to this preference other than the widely known association of *A.* *baumannii* to confined battlefield, which is primarily dominated by men [69]. Thus, more focus vertical analysis with larger sample size is likely to bring more insights into the pathogenesis of this organism. *Acinetobacter* *baumannii* was shown to be the most resistant pathogen isolated from clinical specimens in all four hospitals. Isolates of this species were fully resistant to almost all antibiotics tested, except for amikacin (AK, 61.25%), colistin (CS, 5%), and ertapenem (ETP, 0%). The latter antibiotic was 100% effective in the treatment of this infection. To tigecycline (TGC), 69.23% of isolates were resistant, only 12.82% sensitive, and 17.95% had a relatively higher intermediate response.

Of the 46 clinical isolates of *P. mirabilis* identified, 52% were at ICU from blood, sputum, urine, and wound infections at equal frequencies. *P. mirabilis* was isolated from COVID-19 patients; however, 7% and 28% isolations were made at surgical and medical wards, respectively, and 13% isolations from all other sources. While no isolates were found in young age patients, only 17% were in adults. Nevertheless, 83% of isolates were recorded in old age men (66%) with underlying causes. Many had catheter-associated polymicrobial urinary tract infections accompanied by urolithiasis consistent with the fimbria-mediated molecular mechanisms of pathogenesis in this species [70]. The isolates showed certain degree of resistance to all the antibiotics tested, ranging from 82% to 100%. Unfortunately, the antibiogram of *P. mirabilis* indicated high levels of multidrug resistance patterns with 100% resistance to important drugs and increasing intermediate resistances against antibiotics of choice including carbapenems. This pattern is similar to other reports and would call for immediate intervention and rigorous stewardship plans to limit the spread of carbapenem resistance genes. A four-year surveillance of uropathogens (2013–2016) in the Aseer region of Saudi Arabia revealed that the majority of uropathogens were resistant to antibiotics commonly used in clinical practice. However, in the case of linezolid, daptomycin, and vancomycin the resistances were the lowest. These findings are consistent with earlier reports and these drugs were recommended as revised empirical treatment for UTIs with continuous surveillance of uropathogens [71].

A total of 71 “other Gram-negative bacterial” isolates were included in this category due to low number of isolates from different species. While single isolates were taken from patients, a few isolates in the same patient showed quite distinct disease patterns and were included for future sequencing. *Providencia stuartii* had the highest frequency of isolation with 22 isolates mostly from sputum samples. They showed unique patterns, i.e., high resistance to cephalosporins, potentiated ampicillin, colistin, gentamicin, and carbapenems, and were sensitive to amikacin except for three of them. This pattern was consistent with the first case of carbapenem-resistant *P. Stuartii* isolate in Riyadh from a sputum sample on day 22 of ICU admission [72]. Potential endogenous infectious with *P. Stuartii* was detected from tracheal aspirates, urine, and blood. *P. Stuartii* is among the most common causes of nosocomial urinary tract infections, pneumonia, and wound and bloodstream infections that end with severe patients’ outcomes [73,74,75]. *P. Stuartii* survives well in natural environment and often causes opportunistic infection in residents of long-term care facilities (LTCFs). Emergence of these organisms in the Ha’il region is consistent with the increased visitors and camping trips in the recent years from major cities such as Riyadh. However, the impressive reduction in gastroenteritis incidences and foodborne infections, including salmonellosis, speaks for the cleanliness of the city and strict enforcement of public health measures in the region. The “other Gram-negative bacteria” category showed low frequencies of isolations from diverse departments. High levels of intermediate resistances were observed in these isolates with almost identical patterns of susceptibilities, resistances, and intermediate resistances across the antibiotics tested. In other words, six antibiotics showed effectiveness above 50%, namely, amikacin (AM, 75.4%), piperacillin/tazobactam (TZP, 70%), meropenem (MRP, 65.2%), ertapenem (ETP, 58%), trimethoprim*/sulfamethoxazole (SXT, 56.3%), and levofloxacin (LEV, 56.3%). Similarly, intermediate resistances to six antibiotics were observed, including cefoxitin (FOX), tigecycline (TGC), ciprofloxacin (CIP), cefepime (FEP), imipenem (IMI), and gentamicin (CN). On the other hand, from 50 to over 90% isolates were resistant to nine antibiotics.

Taken together, this current cohort-surveillance study revealed the most dominant drug-resistant bacterial species in four Ha’il hospitals, clinical specimen types involved, and correlations of age and gender differences in susceptibility and highest frequencies of isolations. Based on recent resistance classifications by Magiorakos et al. (2012), *A. baumannii*, *P. aeruginosa*, and enteric bacteria were defined as pan-resistant, extremely resistant, and multi-drug resistant, respectively. In 616 positive isolates, the most frequent bacteria species isolated were *K. pneumoniae* (*n* = 187, 30%), *E. coli* (*n* = 151, 24.5%), *P. aeruginosa*, (*n* = 84, 13.6%), *A.* *baumannii* (*n* = 82, 13.3%), and *P. mirabilis* (*n* = 46, 7%) in that order. *Acinetobacter* *baumannii* (35%) and *K. pneumoniae* (23%) were the dominant coinfection isolates in SARS-CoV2 infections. In addition, significant number of isolates (*n* = 77, 12.5%) not belonging to a specific ward were categorized as “other Gram-negative bacteria” and showed unique species-specific resistance patterns while sharing common Gram-negative profiles. Among these, the carbapenem-resistant *P. stuartii* was identified for the first time in Ha’il with the highest frequency of isolation, particularly in male senior residents of long-term care facilities. Furthermore, the influence of specimen, age, and gender differences on disease patterns and susceptibility was significant. For instance, correlations of specimen sources and age and gender differences has shown the following rates of infections: 30% in ICU, 17.5%in medical wards, and 13.5% in COVID-19 zones, mostly in male (59%) senior (54%) patients. Overall ICU infections were 52% *P. mirabilis*, 49% *A. baumanii*, 41% *P. aeruginosa,* 24% *K. pneumoniae,* and 21% *E. coli*, and most of the respiratory infections were caused by carbapenem-resistant *A. baumanii* and *K. pneumoniae* and UTI by *K. pneumoniae* and *E. coli.* The high level of Gram-negative resistance is challenging especially for geriatric patients in long-term care. The domination of only two bacterial species coinfections in SARS-CoV2 is worth further vertical genomic studies. Continuous monitoring of resistance, stewardship program, and search for novel drugs are recommended. The Gram-negative bacteria resistance rates are not entirely dependent on the local antibiotic usage practices; instead, they are the results of local as well as global multifactorial aspects of transmission dynamics. Saudi Arabia is the largest economic and pilgrimage hub with close social and cultural ties to countries in the region and it is also a significant global partner. This has created extensive human dynamics, particularly during regional conflicts and political unrests. Therefore, the resistance patterns observed are typical profiles in the region and elsewhere, in different geographic locations around the world. Despite these difficulties, the country is able to operate an impressive healthcare system with rigid biocontainment, hospital infection control practices, and resistance monitoring programs. Following global recommendations, several programs are being refined and tuned up regularly, including multipoint surveillances, inter-laboratory networks for data sharing, and, recently, the introduction of whole-genome-based national surveillance programs in the country, which are expected to reduce nosocomial resistances in the near future [76].

## Figures and Tables

**Table 1:** Specimens’ sources, patients’ age and gender, and resistance profiles of major Gram-negative bacteria recovered from four major hospitals in Ha’il, Saudi Arabia.

Ward ^a^	Bacterial Clinical Isolates (*n* = 621)											
	*K. pneumoniae* (*n* = 178)		*E. coli* (*n* = 151)		*P. aeruginosa* (*n* = 84)		*A.baumannii* (*n* = 82)		*P. mirabilis* (*n* = 46)		*Other* *Gram*- *Negatives* (*n* = 71)	
	*n*	%	*n*	%	*n*	%	*n*	%	*n*	%	*n*	%
Intensive Care Unit ICU (30%)	42 Urine (16) Sputum (9) Blood (8) Wound/pus (7) AE (2)	24	31 Urine (16), sputum (6) blood (6), wound/pus (3)	21	34 Urine (5), Blood (5) Sputum (15) Wound swab/pus (2) Other (7)	41	40 Sputum (20) Blood (8) Swab/wound/pus (8) Other (4)	49	24 Blood (7) Sputum (6) Urine (5) Wound/pus/swab (6)	52	15 Urine (3) Blood (2) Sputum (4) Wound/swab/pus (3) Other (3)	21
COVID-19 Isolation Zones (ISO) or Ward (COW) 13.5%	40 Blood (15) Sputum (12) Urine (6) Wound/pus/swab (5) Other (2)	23	7 Blood (1) Sputum (1) Wound (1) Urine (4)	5	0	0	29 Bloody sp. (12) Urine (2) Sputum (11) Wound (4)	35	0	0	2 Blood Wound	3
Surgical Ward (SW) 12%	25 Wound/pus/swab (8) Urine (11) Blood (1) Other (5)	14	19 Wound (7) Urine (8) Blood (1) Other (3)	13	9 Wound (3) Sputum (4) Urine (2)	11	3 Wound (3)	4	3 Swab (2) Sputum (1)	7	13 Sputum (6) Wound (4) Blood (1) Other (2)	18
Male/Female Medical Ward (M/F MW) 17%	21 Blood (3) Wound (3) Urine (11) Sputum (2) Other (2)	12	44 Urine (26) Swabs (9) Blood (2) Sputum (1) Other (5)	29	6 Wound swab (4) Urine (2)	7	5 Wound (1) Urine (1) Other (3)	6	13 Urine (10) Sputum (2) Blood (1)	28	16 Urine (9) Swabs (3) Blood (2) Stool (2)	23
AKU—AMR 4%	8 Blood (4) Other (4)	4.5	3 Urine (3)	2	6	7	0	0	0	0	5 Blood (5)	7
Other (25%)	42	24	47	30	29	35	5	6	6	13	24	34
Total *n* = 616	178	100%	151	100%	84	100%	82	100%	46	100%	75 ^b^	100%
Age ^b^												
Young 1–20 (16%)	14	11	34	26	16	23	5	9	0	0	12	20
Adults (21–49 y) (30%)	43	33	41	32	27	39	15	27	7	17	14	21
Seniors (>50 y) (54%)	74	57	55	42	26	38	35	64	34	83	40	61
Gender ^c^												
Male (59%)	72	53	57	43	59	75	43	74	29	66	45	63
Female (41%)	64	47	75	57	20	25	15	26	15	34	26	37
MDR, XDR, PDR ^d^	MDR		MDR		XDR		PDR		XDR		MDR	

**^a^** overall % of isolates per ward; **^b^** %isolates per age; **^c^** %isolates per gender; **^d^** MDR, multidrug-resistant; XDR, extremely drug-resistant; PDR, pan-drug-resistant.

**Table 2:** Antimicrobial susceptibility patterns of “other Gram-negative bacteria” isolates from various sources in four major Ha’il Hospitals, Saudi Arabia.

Organisms	Gender	Age ^a^	Specimen	Ward ^b^	AK	CN	ETP	IMI	MRP	KF	CXM	FOX	CAZ	CRO	FEP	ATM	AMP	AUG	TZP	CS	SXT	NIT	CIP	LEV	TGC	Hospital
*P. stuartii*	F	71	Swap	FMW	S	S	S	S	S	R	S	S	S	S	S	S	I	R	S	R	S	R	S	S		KKH
*P. stuartii *	F	74	Sputum	ICU	R	R	S	R	R	R	R	S	R	R	R	R	R	R	R	R	R	R	R	R		KKH
*P. stuartii*	M	81	Blood	AE	I	R	S	S	S	R	R	I	S	S	S	S	R	R	S	R	S	R	R	R		KKH
*P. stuartii*	M	31	Sputum2-B	MSW	S	R	S	S	R	R	I	S	S	S	S	S	R	R	S	R	S	R	I	R		KKH
*P. stuartii*	M	38	Wound	ICU	S	R	S	S	S		R	S	R	R	R	R	R	R	R	R	R	R	R	R		KKH
*P. stuartii*	M	68	Sputum	MSW	S	R	S		S	R	R	S	R	R	R	R	R	R	S	R	R	R	R	R		KKH
*P. stuartii*	M	33	Urine	EAB	S	R	R	R	S	R	R	R	R	R	R	R	R	R	S	R	R	R	R	R	X	KKH
*P. stuartii *	M	61	Sputum	ICU	S	R	R	R	R	R	R	S	R	R	R	R	R	R	S	R	R	R	R	R	X	KKH
*P. stuartii *	F	61	Dialysis	ICU	S	R	S	X	X	R	R	R	R	R	R	R	R	R	R	R	R	R	R	R	X	KKH
*P. stuartii*	F	52	Urine	HND	S	R	S	S	R	S	S	S	S	S	S	R	R	S	R	R	R	R	R	R	X	KKH
*P. stuartii*	M	31	Sputum	MSW	S	R	S		R	R	I	S	S	S	S	S	R	R	S	R	S	R	I	R		KKH
*P. stuartii*	F	71	Sputum	ICU	S	R	R		S	R	R	R	R	R	S	R	R	R	S	R	R	R	R	R		KKH
*P. stuartii*	F	52	Urine	LAB	R	R	S		S	R	R	S	R	R	R	R	R	R	S	R	R	R	R	R		KKH
*P. stuartii*	F	42	Wound	FMW	S	S	S	X	S	R	S	S	S	S	S	S	S	R	S	R	S	R	I	I		KKH
*P. stuartii*	M	40	Wound		S	R	R	X	R	R	R	S	R	S	S	R	R	R	R	R	R	R	R	R	R	KSSH
*P. stuartii*	M	6	Sputum	MSW	S	S	S		S	R	S	S	R	R	R	S	R	S	S	R	R	R	R	R		KKH
*P. stuartii*	M	40	Sputum	MSW	S	S	R	S	S	R	S	R	R	S	R	R	R	S	S	R	S	R	S	R	X	KKH
*P. stuartii*	M	51	Other	LAB	S	S	R	X	S	R	R	S	S	R	R	R	S	R	S	R	S	R	R	S	X	KKH
*P. stuartii*	F	65	Urine	ICU	S	R	S	R	R	R	R	S	R	S	R	R	S	R	R	R	R	R	S	R	X	KKH
*P. stuartii*	F	52	Urine	HND	S	R	S	S	R	S	S	S	S	S	S	R	R	S	R	R	R	R	R	R	X	KKH
*P. stuartii*	F	30	Sputum	MSW	S	R	S		R	S	I	S	R	S	R	S	R	R	S	R	S	S	R	R		KKH
*P. stuartii*	M	72	Wound		S	R	R	X	S	R	R	S	S	S	S	S	R	R	S	R	S	R	S	S	R	KSSH
*M. morganii*	M	54	Pus	MSW	S	S	S		S	R	R	S	R	R	S	S	R	R	S	R	R	I	I	I		
*M. morganii*	M		AE–AN	ICU	S	S	S	S	S	R	R	S	S	S	S	S	R	R	S	R	R	R	S	S	R	
*M. morganii*	M	61	Blood	ICU	S	R	S	S	S	R	R	S	R	R	R	I	R	R	S	R	R	R	R	R	R	
*M. morganii*					S		S	S			S	S	S		S	S	R	S	S		S		S	S	s	MCH
*M. morganii*	F	33			S	S	S	X	S	R	R	S	S	S	S	S	R	R	S	R	S	R	S	S	S	KKH
*M. morganii*	F	54	Wound	FSW	S	S	S	X	S	R	R	S	S	S	S	S	R	R	S	R	S	R	S	S	R	KKH
*M. morganii*	M	51	Blood	MSW	S	S	S	X	S	R	R	S	R	R	R	S	R	R	S	R	S	R	S	S	R	KKH
*M. morganii*	F	79	Urine		S	R	R	R	S	R	R	I	S	R	R	R	R	R	S	R	R	R	R	R	R	KSH
*S. fonticola*	M	85	Urine	MMW	S	S	R	I	R	R	R	S	S	R	R	R	R	S	S	S	S	R	S	S	R	
*S. fonticola*	M	88	Urine	MMW	S	S	R	R	R	R	R	R	S	S		S	R	R	S	R	R	R	S	S	R	
*Haemophilus* sp.	M	3m	Eyes swap	PICU	R	S	S	S	R	S	S	S	S	S	S	S	R									
*C. koseri*	F	62	Blood	AKU	S	S	S	S	S	S	R	S	S	S	S	S	R	S	S	S	S	R	S	S	S	
*C. koseri*	F	28		FSW	S	S	S	S	S	S	S	S	S	S	S	S	R	S	S	S	S	S	S	S	S	KKH
*C. koseri*	M	3m	Urine	PW	S	S	R	S	S	R	R	S	R	R	R	R	R	S	S	S	S	R	S	S	R	
*C. koseri*	M	8M	Urine	PICU	R	I	X	I	S	R	R	S	R	R	R	R	R	S	S	R	R	I	S	S	I	KKH
*K. oxytoca*	M	3	Urine	PW	S	S	R	S	S	R	R	R	R	R	R	S	R	S	S	S	R	S	S	S	S	
*K. oxytoca*	M	6M	Urine	UR	S	S	S	S	S	S	S	S	S	S	S	S	R	S	S	S	S	R	S	S	S	
*K. oxytoca*	M	7M	Urine	UPO	S	S	S	S	S	S	S	S	S	S	S	S	R	S	S	S	S	S	S	S	S	KKH
*K. oxytoca*	M	96	Urine	LAB	S	S	R	R	R	R	R	S	R	R	R	R	R	R	R	S	S	S	S	S	S	KKH
*K. ozae*	M	64	Urine		R	R	R	R	R	R	R	R	R	R	R	R	R	R	R	R	R	R	R	R	S	KSSH
*S. enterica*			Urine	AE/ER			S	S	S				S		S	S	S	S	s		s		S	S	s	MCH
*S. typhi*							S	S	S				S		S	S	S	S	S		S		S	S	S	
*Salmonella* sp.	M	4M	Stool	PW	S	S	S	S	S	S	S	S	S	S	S	S	S	S	S	S	S	S	S	S	S	KKH
*P. fluorescens*	M	24	Blood	MMW	S	I	S	S	S	R	R	R	S	R	S	R	R	R	S	S	S	R	I	S	S	KKH
*P. putida*	F	65	Urine	FMW	S	S	R	S	S	R	R	R	S	S	I	I	R	R	S	S	R	R	I	R	R	KKH
*S. maltophilia*	F	18	Wound	FSW	R	R	R	R	R	R	R	R	R	R	R	R	R	R	R		S	R	R	S	I	KKH
*S. maltophilia*	M	70	Blood	AMR	R	R	R	R	R	R	R	R	R	R	R	R	R	R			S	R	I	S	I	KKH
*S. maltophilia*	M	64	Blood	AMR	R	R	R	R	R	R	R	R	R	R	R	R	R	R	R	X	S	R	I	S	I	KKH
*S. maltophilia*	M	64	Blood	MMW	R	R	R	R	R	R	R	R	R	R	R	R	R	R	R		S	R	S	S	S	KKH
*S. maltophilia*	M	70	Blood	AMR	R	R	R	R	R	R	R	R	R	R	R	R	R	R	R	X	S	R	R	S	S	KKH
*S. maltophilia*	M	45	Bloody Sputum		R	R	R	R	R	R	R	R	R	R	R	R	R	R	R	R	R	R	R	S	S	KSSH
*P. rettgeri*	F	61	Blood	ICU	S	I		I	S	R	S	S	S	S	S	S	R	R	S	R	S	R	S	S		KKH
*P. rettgeri*			Wound		S	S	S	I	S	R	S	S	S	S	S	S	R	R	S	R	S	S	S	S	R	KSSH
*P. rettgeri*	M	70	Wound	MSW	S	R	S	R	S	R	R	S	R	R	R	R	R	R	S	R	R	R	R	R	R	KKH
*C. freundii	M	56	Blood	COW	R	R	R	R	R	R	R	R	R	R	R	R	R	R	R	S	S	S	R	S	S	KKH
*C. freundii*	M	65	Wound	COW	R	R	R	R	R	R	R	R	R	R	R	R	R	R	R	R	R	S	R	R	S	KKH
*C. freundii*	M	2M	Urine	PW	S	R	S	S	S	R	R	R	R	R	R	R	R	R	S	S	S	S	S	S	S	KKH
*C. freundii*	F	81	Wound	ICU	S	S	S	S	S	R	R	R	R	R	R	R	R	R	R	S	R	S	R	R	S	KKH
*C. freundii*	F	62	wound	FMW	S	R	S	S	S	R	R	R	R	R	R	R	R	R	S	S	S	S	S	S	S	KKH
*C. freundii*	M	75		ICU	S	R	S	R	S	R	R	R	R	R	R	R	R	R	I	S	R	S	R	R	S	KKH
*C. koseri*	F	62	Blood	AKU	S	S	S	S	S	S	R	S	S	S	S	S	R	S	S	S	S	R	S	S	S	
*C. koseri*	F	28		FSW	S	S	S	S	S	S	S	S	S	S	S	S	R	S	S	S	S	S	S	S	S	KKH
*C. koseri*	M	3m	Urine	PW	S	S	R	S	S	R	R	S	R	R	R	R	R	S	S	S	S	R	S	S	R	
*C. koseri*	M	8M	Urine	PICU	R	I	X	I	S	R	R	S	R	R	R	R	R	S	S	R	R	I	S	S	I	KKH
*B. cepacia*	F	49	Urine	FMW	R	R	R	R		R	R	R	S	R		R	R	R	S	R	S	R	R	R	S	
*B. cepacia*	M	63	Sputum	ICU	S	S	R	I		R	R	R	R	R		R	R	R	R	R	R	R	R	R	S	
*P. vulgaris*	M	66	Urine		R	R	I	R	I	R	R	I	R	R	R	R	R	R	I	R	S	R	R	R	R	KHH
*Shigella* sp.	M		Stool	LAB		R	S	S	S				S		S	S	R	S	S		S		S	S	S	MCH
*Shigella* sp.	M		Stool	LAB	S		S	S	S				S		S	S	I	S	S		S		S	S	S	MCH
*Shigella* sp.	F		Stool	FMW		R	S	S	S				S		S	S	R	S	S		S		S	S	S	KKH
*Shigella* sp.	F		Stool	LAB	S	R	S	S	R				S		R	S	I	S	R		S		S	I	S	KKH
*C. lapagei*	M	58	Urine	MMW	R	R	R	R	R	R	R	R	R	R	R	R	R	R	S	R	R	R	R	R	S	KKH
*C. werkmanii*	F	61	Urine	LAB	S	S	S	S	S	R	S	R	S	S	S	S	R	R	S	S	S	S	S	S	S	KKH

Footnotes: ^a^ Age color code: red = seniors; brown = infants and children below 18; blue = young adults and middle-aged patients. ^b^ Ward color code: red = ICU, PICU—pediatric ICU; blue = MMW—men’s medical ward, FMW—female medical ward; green = FSW—female surgical ward, AE/ER—accidents and emergency, LAB—laboratory, UR—urology, UPO—outpatient, EAB—enhanced assessment beds, COW—COVID-19 isolation ward; AMR—AKU male room. KKH—King Khalid Hospital, MCH—Maternity and Children Hospital, KSSH—King Salman Specialist Hospital.

## Data Availability

All data used in the study is reported in the manuscript directly.
